# Recadastramento da população residente em Foz do Iguaçu, Brasil, em atendimento à Política de Atenção Primária à Saúde

**DOI:** 10.26633/RPSP.2022.158

**Published:** 2022-12-28

**Authors:** Marília Miranda Forte Gomes, Rebeca Carmo de Souza Cruz, Ana Maria Nogales Vasconcelos, Adriana Izuka, Carmensita Gaievski Bom, Larissa Djanilda Parra da Luz, Kátia Yumi Uchimura, Sandro Terabe, Juan José Cortez-Escalante, Maria Almirón

**Affiliations:** 1 Universidade de Brasília (UnB) Brasília (DF) Brasil Universidade de Brasília (UnB), Brasília (DF), Brasil.; 2 Secretaria Municipal de Saúde de Foz do Iguaçu Foz do Iguaçu (PR) Brasil Secretaria Municipal de Saúde de Foz do Iguaçu, Foz do Iguaçu (PR), Brasil.; 3 Organização Pan-Americana da Saúde/Organização Mundial da Saúde (OPAS/OMS). Organização Pan-Americana da Saúde/Organização Mundial da Saúde (OPAS/OMS).

**Keywords:** Estatísticas de população, atenção primária à saúde, saúde na fronteira, Brasil, Populations characteristics, primary health care, border health, Brazil, Características de la población, atención primaria de salud, salud fronteriza, Brasil

## Abstract

**Objetivo.:**

Apresentar a experiência e os resultados do recadastramento da população residente em Foz do Iguaçu, um município de fronteira localizado no estado do Paraná, Brasil, para atender às diretrizes da Política de Atenção Primária à Saúde (APS) e ao seu novo modelo de financiamento pelo Programa Previne Brasil.

**Métodos.:**

Utilizando uma estratégia de varredura (amostra de conveniência) para coleta de dados, foram visitados 52 263 domicílios e realizadas 22 710 entrevistas de setembro a novembro de 2019. As entrevistas foram realizadas pessoalmente por 54 agentes comunitários de saúde. Foram coletados dados sobre o domicílio (*status* de posse da moradia, localização urbana ou rural, tipo de domicílio, material da construção, disponibilidade de rede elétrica e de esgoto, abastecimento de água e destino do lixo) e informações demográficas e de saúde dos moradores.

**Resultados.:**

O recadastramento revelou que os domicílios eram predominantemente casas próprias, em área urbana, bem edificados e servidos por energia elétrica, rede geral de água e coleta de lixo. Sobre a população recadastrada, 52,8% eram mulheres, 62,5% tinham idade de 15 a 59 anos e 60,0% se autodeclararam brancos. Entre os entrevistados com 15 anos ou mais, 90,0% tinham completado o ensino fundamental. A principal ocupação foi “assalariado com carteira de trabalho”. Ainda, 18,6% dos entrevistados se autodeclararam hipertensos e 7,0%, diabéticos.

**Conclusões.:**

O recadastramento trouxe informações relevantes para subsidiar o planejamento da APS, assim como iniciativas de assistência social, trabalho e habitação; também foi fundamental para definir estratégias de atenção à saúde nesse município de fronteira durante a pandemia de COVID-19.

## Abstract (en)

**Objective.:**

To present the experience and results of the reregistration of residents in Foz do Iguaçu, a border town located in the state of Paraná, Brazil, to meet the guidelines of the national Primary Health Care (PHC) Policy and its new financing model (*Programa Previne Brasil*).

**Method.:**

A scanning strategy (convenience sample) was used for data collection, with 52 263 households visited and 22 710 interviews conducted from September to November 2019. The interviews were conducted face-to-face by 54 community health workers. Data were collected on the household (ownership status, urban or rural location, type of household, construction material, availability of electrical and sewage networks, water supply and waste disposal). Demographic and health information on the residents was also collected.

**Results.:**

The reregistration process revealed that most residents were home owners and lived in well-constructed homes, located mostly in urban areas, served by electricity, with access to water supply and garbage collection. Of the reregistered population, 52.8% were women, 62.5% were aged between 15 and 59 years and 60.0% declared themselves white. Among respondents aged 15 or over, 90.0% had completed elementary school. The main occupation was “formal salaried job”. Additionally, 18.6% of the interviewees declared themselves to be hypertensive and 7.0%, diabetic.

**Conclusions.:**

The reregistration process uncovered relevant information to support both PHC planning as well as social assistance, work and housing initiatives; it was also fundamental to define health care strategies in this border town during the COVID-19 pandemic.

## RESUMEN

**Objetivo.:**

Presentar la experiencia y los resultados de la reinscripción de la población residente en Foz do Iguaçu, un municipio fronterizo ubicado en el estado de Paraná (Brasil), en cumplimiento de las directrices de la Política de atención primaria de salud y su nuevo modelo de financiamiento (Programa Previne Brasil).

**Métodos.:**

Utilizando una estrategia de barrido (muestreo de conveniencia) para la recolección de datos, se visitaron 52 263 hogares y se realizaron 22 710 entrevistas entre septiembre y noviembre de 2019. Las entrevistas fueron presenciales y estuvieron a cargo de 54 trabajadores comunitarios de salud. Se recopilaron datos sobre el hogar (régimen de propiedad de la vivienda, ubicación en una zona urbana o rural, tipo de vivienda, material de construcción, disponibilidad de redes de energía eléctrica y alcantarillado, abastecimiento de agua y eliminación de desechos) e información sobre la composición demográfica y la salud de los residentes.

**Resultados.:**

La reinscripción reveló que los residentes eran propietarios de sus viviendas y que estas se encontraban ubicadas en zonas urbanas, estaban bien construidas y tenían servicios de energía eléctrica, abastecimiento de agua y recolección de basura. El 52,8% de la población registrada correspondió a mujeres, el 62,5% tenía entre 15 y 59 años y el 60,0% declaró que era de raza blanca. El 90,0% de los entrevistados mayores de 15 años había terminado la escuela primaria. La ocupación principal era “persona asalariada con carnet de trabajo”. Además, el 18,6% de los entrevistados indicó que tenía hipertensión y el 7,0%, diabetes.

**Conclusiones.:**

La reinscripción aportó información relevante para apoyar la planificación de la atención primaria de salud , así como las iniciativas en materia de asistencia social, trabajo y vivienda; también fue fundamental para definir las estrategias de atención de salud en ese municipio fronterizo durante la pandemia de COVID-19.

Um dos grandes desafios da gestão em saúde nos municípios brasileiros é a produção de conhecimento com vistas a aprimorar processos de trabalho, proporcionar adequada atenção à saúde para a população e contribuir para que o Sistema Único de Saúde (SUS) seja equitativo, universal e integral nos âmbitos local, regional e nacional. O Ministério da Saúde (1) ressalta a relevância da informação oportuna, detalhada e de qualidade para a gestão descentralizada do SUS quando afirma que é desejável que o município “seja capaz de produzir, organizar e coordenar a informação em saúde de sua região” (p. 166). Nesse sentido, é importante que os municípios brasileiros desenvolvam capacidade técnica para que, além de coletar dados para alimentar os Sistemas de Informações em Saúde (SIS), possam utilizar esses mesmos dados para produzir conhecimento relevante para a gestão. Deve-se considerar, por outro lado, que dados dos SIS podem ser insuficientes para o planejamento municipal na área da saúde. A gestão municipal deve identificar as lacunas de informação, assim como a metodologia a ser utilizada para a coleta e análise de dados, tendo em vista a melhor adequação dos serviços à demanda existente.

**FIGURA 1.**
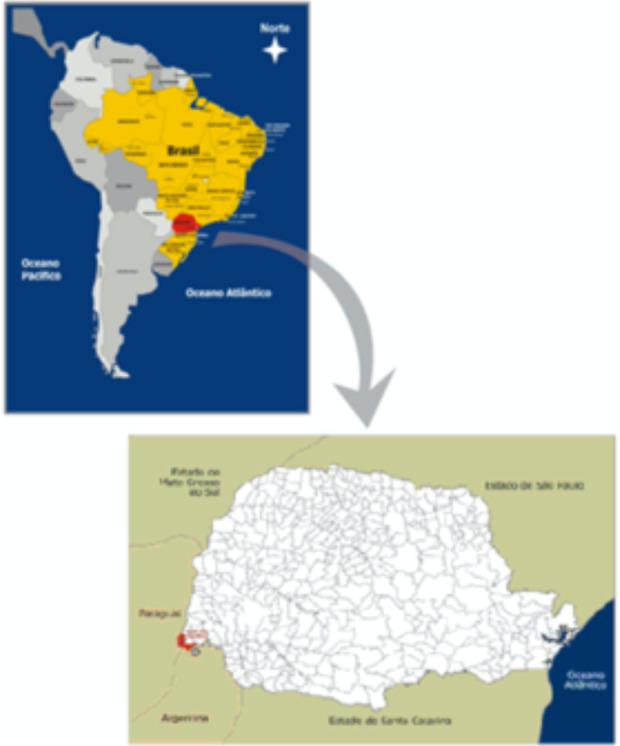
Localização de Foz do Iguaçu, estado do Paraná, Brasil

Quando há necessidade de proposição de ações específicas, diante de cenários particularmente complexos, por vezes é necessário um maior detalhamento sobre o perfil do usuário do sistema público de saúde. Esse é o caso de Foz do Iguaçu, município localizado na tríplice fronteira Brasil, Argentina e Paraguai (figura 1), caracterizado por intenso fluxo migratório, comercial e turístico. Assim como outros municípios fronteiriços no Brasil, Foz do Iguaçu possui uma demanda elevada por serviços públicos de saúde por parte de uma população que não tem residência habitual no município.

Em 2019, o governo federal instituiu o Programa Previne Brasil, que estabeleceu um novo modelo de financiamento para a APS no SUS (2). Conforme esse modelo, os incentivos financeiros repassados aos municípios são calculados com base na população cadastrada na equipe de Saúde da Família (eSF) e na equipe de Atenção Primária (eAP) no Sistema de Informação em Saúde para a Atenção Básica (SISAB); na vulnerabilidade socioeconômica da população cadastrada na eSF e na eAP; no perfil demográfico por faixa etária da população cadastrada na eSF e na eAP; e na classificação geográfica definida pelo Instituto Brasileiro de Geografia e Estatística (IBGE). Portanto, conhecer detalhadamente a condição de residência dos usuários e o tipo de atendimento buscado é uma ação estratégica para garantir que os recursos alocados sejam ajustados ao atendimento à saúde de fato prestado por esses municípios.

Desse modo, realizou-se, de setembro a novembro de 2019, o recadastramento da população residente em Foz do Iguaçu no âmbito da cooperação técnica entre a Organização Pan-Americana da Saúde (OPAS) e a Secretaria Municipal de Saúde (SMSA). Entre os objetivos desse recadastramento, destacamse: atualizar e aprimorar os indicadores básicos de saúde do município; ampliar a população cadastrada na ESF (3); e obter subsídios para uma revisão das estimativas de população residente no município. Após o recadastramento, a grave crise sanitária imposta pela covid-19 evidenciou a importância dos sistemas universais de atenção à saúde da população, especialmente o papel da APS na prevenção e no controle da pandemia (3, 4).

Nesse contexto, o presente artigo tem como objetivo apresentar a experiência e os resultados do recadastramento da população residente em Foz do Iguaçu para atender às diretrizes da Política de APS e ao novo modelo de financiamento pelo Programa Previne Brasil.

***Fonte:*** Secretaria Municipal de Saúde de Foz do Iguaçu.

## MATERIAIS E MÉTODOS

Trata-se de estudo descritivo, tipo relato de experiência, sobre a ação de recadastramento (Cadastre Já) da população de Foz do Iguaçu no e-SUS. O recadastramento foi realizado pela SMSA em parceria com a OPAS nos meses de setembro a dezembro de 2019 (5). Foi realizado um levantamento de base populacional e domiciliar com o objetivo de obter dados sobre os perfis sociodemográfico e epidemiológico da população residente. Foz do Iguaçu é o município fronteiriço brasileiro com maior volume populacional, estimado em cerca de 260 mil habitantes em 2020.

### Coleta de dados

O recadastramento foi realizado por meio de visitas a domicílios particulares e operacionalizado por meio de varredura simultânea (amostra de conveniência) dos cinco distritos sanitários de Foz do Iguaçu (Norte, Nordeste, Leste, Oeste e Sul). O recadastramento observou a organização territorial da APS do município, com cinco níveis de hierarquia: distrito sanitário, área, microárea, quarteirão e domicílio (6). Um levantamento censitário de imóveis realizado pelo Centro de Controle de Zoonose (CCZ) do município de Foz do Iguaçu em 2018 e a malha cartográfica municipal atualizada permitiram construir uma base cadastral de domicílios a serem visitados. Foram excluídos os domicílios coletivos com população institucionalizada (domiciliados em asilos, hospitais, presídios).

A coleta por varredura não constitui uma amostra aleatória da população do município. Ocorreram perdas correspondentes aos domicílios fechados visitados mais de duas vezes em períodos diferentes e às recusas, quando expressas pessoalmente, após visita ao domicílio e tentativa de entrevista. Com base no levantamento de imóveis realizado pelo CCZ, observou-se uma sobrerrepresentação do distrito Nordeste e uma sub-representação do distrito Oeste (tabela 1). No total, foram visitados 52 263 domicílios (51,6% dos domicílios cadastrados pelo CCZ) e realizadas 22 710 entrevistas, correspondendo a uma taxa de resposta de 43,5%.

As entrevistas foram realizadas pessoalmente por 54 agentes comunitários de saúde (ACS) utilizando um assistente digital (*personal digital assistant*, PDA). Utilizou-se o aplicativo Epicollect 5 para a coleta e a geração de banco de dados, no qual, previamente, haviam sido inseridos todos os dados de localização dos domicílios levantados pelo CCZ. Esses dados de localização referem-se às numerações de áreas, microáreas e quarteirões, o que facilitou a organização do trabalho de campo.

O questionário teve duas partes, sendo a primeira destinada ao cadastro do domicílio e a segunda dedicada ao levantamento de informações demográficas e de saúde dos moradores por família existente em cada domicílio visitado. Cabe destacar que o instrumento utilizado foi baseado nos cadastros domiciliar e individual do e-SUS (7).

### Características levantadas

As características relacionadas às condições de moradia incluíram situação de moradia (próprio, já pago; próprio, ainda pagando; alugado; cedido; arrendado; ocupação ou outros), localização (urbana, rural), tipo de domicílio (casa, apartamento, cômodo ou outro), material predominante (alvenaria/taipa com revestimento, alvenaria/taipa sem revestimento, madeira aparelhada, material aproveitado, palha ou outro material), disponibilidade de rede elétrica, abastecimento de água (rede encanada, poço/nascente, cisterna ou outro), forma de escoamento do banheiro ou sanitário (rede coletora de esgoto ou pluvial; fossa séptica; outras formas, como fossa rudimentar, direto para um rio, lago ou mar a céu aberto; ou outra forma) e destino do lixo (coletado, queimado/enterrado, céu aberto ou outro).

No que se refere às características sociodemográficas, foram levantadas para todos os moradores do domicílio as seguintes informações: idade (em anos), sexo (feminino, masculino), raça/cor (branca, preta, parda, amarela ou indígena), escolaridade mais elevada (analfabeto, alfabetizado, fundamental, médio ou superior), nacionalidade (brasileiro, estrangeiro) e situação no mercado de trabalho (para os ativos, foi detalhada a posição na ocupação; para os inativos, identificou-se se era aposentado ou pensionista).

As características relacionadas às condições e situações de saúde autorreferidas dos moradores dos domicílios visitados incluíram ter plano privado de saúde (sim, não), estar gestante (sim, não), estar acamado (sim, não) e ter diagnóstico de hipertensão (sim, não) e diabetes (sim, não).

Todas as análises foram realizadas com os programas estatísticos SPSS 21 e RStudio (versão 1.2.1335).

**TABELA 1.** Domicílios visitados para recadastramento segundo levantamento, tipo de entrevista e distrito, Foz do Iguaçu (PR), Brasil, 2019

Distrito	Recadastramento em 2019				CCZ^a^	Domicílios visitados(%)^b^
	Sim	Não	Total	Taxa de sucesso (%)		
Leste	6 933	8 372	15 305	45,3	27 639	55,4
Nordeste	5 740	5 335	11 075	51,8	13 376	82,8
Norte	4 909	9 466	14 375	34,1	26 154	55,0
Oeste	1 344	2 112	3 456	38,9	21 578	16,0
Sul	3 784	4 268	8 052	47,0	12 445	64,7
Total	22 710	29 553	52 263	43,5	101 192	51,6

***Fonte:*** Secretaria Municipal de Saúde de Foz do Iguaçu, Cadastre Já, 2019.

a CCZ: Centro de controle de zoonoses. Em 2018, o CCZ fez um levantamento censitário de imóveis em Foz do Iguaçu, que serviu como base cadastral para selecionar os domicílios a serem visitados.

b Porcentagem relativa ao total de domicílios incluídos no levantamento censitário do CCZ.

### Aspectos éticos

A experiência da SMSA em parceria com a OPAS relatada neste artigo refere-se ao recadastramento da população adstrita pelas unidades básicas de saúde, atendendo à Política de APS. A visita aos domicílios e o levantamento de dados sobre os residentes fazem parte da rotina de serviços dos ACS. O armazenamento e a organização dos dados coletados são de responsabilidade da SMSA, como ocorre com outros registros administrativos. Por se tratar de uso de base de dados com informações agregadas, sem a possibilidade de identificação individual, o estudo dispensa aprovação por comitê de ética em pesquisa (8).

## RESULTADOS

O recadastramento evidenciou a característica essencialmente urbana do município de Foz do Iguaçu, com 99,8% dos domicílios entrevistados localizados em área urbana (tabela 2). No que se refere à condição de ocupação do imóvel de residência, o imóvel era próprio em 77,8% dos domicílios e, em 17,1%, era alugado ou arrendado. A partir do detalhamento por distritos, observou-se que, no distrito Oeste, o percentual de imóveis alugados era mais elevado (28,7%), muito superior à proporção média encontrada. Outra situação que merece destaque foi a elevada proporção de imóveis na categoria “ocupação” no distrito Sul — a mais elevada no município (6,7%).

Com relação ao tipo do imóvel, 96,6% eram casas, embora mereça destaque a área central do município (distrito Oeste), onde a verticalização das habitações foi mais frequente (22,8%). Quanto ao material predominante, a edificação era de boa qualidade em 95,4% dos domicílios entrevistados, sendo 90,4% de alvenaria com revestimento e 5,0% de madeira aparelhada (madeira lisa). Nos distritos Oeste e Sul, houve maior frequência do tipo de edificação com madeira aparelhada (11,1% e 7,2%, respectivamente). No distrito Sul, também os materiais que denotam precariedade da construção tiveram maior frequência (5,2% de alvenaria sem revestimento e 3,9% de taipa, palha ou outro material).

No que se refere aos serviços urbanos (tabela 2), mais de 95% dos domicílios entrevistados eram atendidos pelos serviços de energia elétrica, abastecimento pela rede geral de água e coleta de lixo. Essas elevadas proporções, com pouca variação entre os distritos, indicam que esses serviços podem ser considerados universais no município. Por outro lado, apenas 76,2% dos domicílios declararam ter escoamento de esgoto ligado à rede geral coletora, sendo esse percentual muito variável entre os distritos (destaca-se o distrito Norte, com menos de 60%).

### Perfil sociodemográfico da população

Na tabela 3, são apresentadas as características da população por distrito, segundo variáveis demográficas e socioeconômicas. Da população recadastrada, 52,8% eram do sexo feminino e 62,5% possuíam idade de 15 a 59 anos. Se, por um lado, a distribuição por sexo foi semelhante para todos os distritos observados, por outro a distribuição etária não foi homogênea: a proporção de pessoas com menos de 15 anos foi maior nos distritos Sul e Nordeste (23,8% e 22,9%, respectivamente). Por sua vez, o distrito Oeste apresentou a estrutura etária mais envelhecida, com proporção de 20,6% de pessoas com 60 anos ou mais, muito superior aos demais distritos.

**TABELA 2.** Características dos domicílios conforme distrito sanitário, Foz do Iguaçu (PR), Brasil, 2019

Variável	% por distrito^a^					Total
	Leste	Nordeste	Norte	Oeste	Sul	
Localização						
Rural	0,0	0,3	0,3	0,1	0,1	0,2
Urbana	100,0	99,7	99,7	99,9	99,9	99,8
Condição da ocupação						
Próprio/financiado	78,5	78,2	79,2	66,0	78,2	77,8
Alugado/arrendado	18,3	17,1	15,8	28,7	12,4	17,1
Cedido	3,0	3,8	4,6	3,7	2,6	3,5
Ocupação	0,1	0,9	0,3	1,2	6,7	1,5
Outro	0,2	0,0	0,1	0,5	0,0	0,1
Tipo de domicílio						
Apartamento	1,6	1,7	1,9	22,8	1,1	2,9
Casa	98,1	97,9	97,9	75,6	97,7	96,6
Cômodo/outro	0,3	0,4	0,2	1,6	1,2	0,5
Material predominante						
Alvenaria/tijolo com revestimento	94,3	90,7	91,4	84,0	83,7	90,4
Alvenaria/tijolo sem revestimento	1,5	3,1	3,2	2,7	5,2	3,0
Madeira aparelhada	3,2	4,8	4,5	11,1	7,2	5,0
Taipa/palha/outro	0,9	1,3	0,9	2,2	3,9	1,6
Serviços urbanos						
Energia elétrica	97,4	95,7	94,0	96,1	96,8	96,1
Água – rede geral	98,4	95,8	95,1	97,4	98,0	96,9
Esgoto – rede coletora	84,3	83,2	56,5	85,0	72,5	76,2
Lixo coletado	98,9	98,0	96,5	96,5	98,4	98,0
No. total	6 891	5 693	4 838	1 325	3 740	22 487

***Fonte:*** Secretaria Municipal de Saúde de Foz do Iguaçu, Cadastre Já, 2019.

a Para o cálculo dos percentuais, foram desconsiderados os domicílios com dados ignorados em cada uma das variáveis.

**TABELA 3.** Características demográficas e socioeconômicas da população conforme distrito sanitário, Foz do Iguaçu, Brasil, 2019

Variável	% por distrito^a^					Total
	Leste	Nordeste	Norte	Oeste	Sul	
Sexo						
Feminino	52,6	52,5	53,5	53,1	52,6	52,8
Masculino	47,4	47,5	46,5	46,9	47,4	47,2
No. total	20 516	17 709	13 313	3 810	11 704	67 052
Grupo etário (anos)						
< 15	19,6	22,9	21,0	17,9	23,8	21,4
15-59	63,1	63,3	61,9	61,5	61,2	62,5
≥ 60	17,4	13,8	17,2	20,6	15,0	16,2
No. total	20 509	17 696	13 293	3 809	11 702	67 009
Raça/cor						
Branca	59,6	61,6	57,3	69,7	58,4	60,0
Parda	35,0	34,1	37,6	25,8	36,0	34,9
Preta	4,7	3,8	4,6	3,5	5,2	4,5
Indígena	0,0	0,0	0,1	0,0	0,1	0,0
Amarela	0,6	0,4	0,5	0,9	0,3	0,5
No. total	20 516	17 709	13 313	3 810	11 704	67 052
Escolaridade						
Nenhuma	8,3	12,0	9,4	7,5	11,2	10,0
Fundamental	41,3	47,3	42,4	34,2	48,0	43,9
Ensino médio	34,8	31,8	30,4	35,7	33,0	32,9
Ensino superior	15,6	8,8	17,8	22,6	7,8	13,3
No. total	19 669	17 103	12 718	3 711	11 167	64 368
Nacionalidade						
Brasileira (Foz do Iguaçu)	44,8	45,5	46,0	39,9	51,9	46,2
Brasileira (outro município)	52,5	51,4	50,7	52,7	45,1	50,5
Estrangeira	1,8	1,1	2,0	4,5	2,3	1,9
Naturalizado	1,0	2,0	1,4	2,9	0,8	1,4
No. total	20 516	17 709	13 313	3 810	11 704	67 052
Indicadores de atividade econômica						
Taxa de atividade	63,4	65,6	60,2	60,7	61,3	62,8
Taxa de ocupação	86,9	83,4	83,8	83,1	87,0	85,2
Taxa de desemprego	13,1	16,6	16,2	16,9	13,0	14,8
No. total	19 669	17 103	12 718	3 711	11 167	64 368
População economicamente ativa						
Assalariado com carteira assinada	40,7	44,5	42,0	34,9	49,0	43,0
Assalariado sem carteira assinada	5,0	4,6	5,2	5,2	6,1	5,1
Autônomo com previdência social	14,0	12,0	14,4	15,6	12,5	13,4
Autônomo sem previdência social	26,3	29,6	23,1	27,7	24,1	26,3
Empregador	4,0	3,3	2,9	7,9	2,1	3,5
Servidos público/militar	7,0	4,6	9,0	6,2	4,1	6,2
Outro	3,0	1,3	3,4	2,5	2,0	2,4
No. total	19 669	17 103	12 718	3 711	11 167	64 368

***Fonte:*** Secretaria Municipal de Saúde de Foz do Iguaçu, Cadastre Já, 2019.

a Para o cálculo dos percentuais, foram desconsiderados os domicílios com dados ignorados em cada uma das variáveis. Para as variáveis de escolaridade, indicadores de atividade econômica e população economicamente ativa, considerou-se a população com 15 anos ou mais.

Com relação a raça/cor, 60,0% da população residente se declararam brancos, 34,9% se declararam pardos e 4,5% se declararam pretos. As categorias de raça/cor indígena e amarela apresentaram proporções inferiores a 1%. Chama a atenção a mais elevada proporção de residentes que se declararam de raça/cor branca ou amarela no distrito Oeste (69,7% e 0,9%, respectivamente). O distrito Norte teve a maior proporção de residentes que se declararam como pardos (37,6%) e o distrito Sul a maior proporção de residentes de raça/cor preta (5,2%).

Para a população com 15 anos ou mais (tabela 3), avaliou-se o nível de escolaridade mais elevado alcançado. No geral, a maioria da população tinha nível fundamental ou médio (43,9% e 32,9%, respectivamente). Chama a atenção a proporção da população sem nenhuma escolaridade nos distritos Nordeste (12%) e Sul (11,2%). Por outro lado, os distritos Oeste (22,6%) e Norte (17,8%) se destacaram pela alta proporção da população com escolaridade superior.

O recadastramento também levantou informações sobre a nacionalidade e a naturalidade dos indivíduos. Verificou-se uma proporção de 96,7% de residentes brasileiros, 1,9% de estrangeiros e 1,4% de naturalizados. O distrito Oeste apresentou o maior percentual de população estrangeira residente (4,5%). Dada a história recente de ocupação do município, a proporção de brasileiros não nativos em Foz do Iguaçu é elevada (50,5%), variando de aproximadamente 53% nos distritos Oeste e Leste a 45,1% no Sul.

Por fim, com base nos dados sobre a atividade econômica para a população com 15 anos ou mais de idade, foram calculados os indicadores taxa de atividade, taxa de ocupação e taxa de desemprego. A taxa de atividade do município é de 62,8%. Essa participação na atividade econômica varia segundo distritos. As taxas mais elevadas encontram-se nos distritos Nordeste e Leste, com 65,6% e 63,4%, respectivamente. Os demais distritos têm taxas de atividade em torno de 60%. Com relação à efetiva participação no mercado de trabalho, observou-se que a taxa de desemprego (taxa de ocupação é o seu complemento) é elevada (14,8%) e varia segundo distrito. Nos distritos Oeste, Nordeste e Norte, as taxas de desemprego são muito elevadas, superiores a 16,0%. Já nos distritos Leste e Sul, a taxa de desemprego foi de 13,1% e 13%, respectivamente. Vale ressaltar que, entre as pessoas que exerciam atividade econômica, a principal ocupação era “assalariado com carteira de trabalho” (43,0% dos ocupados). Em segundo lugar estava a posição “autônomo sem previdência social”, com 26,4% dos ocupados. A distribuição foi semelhante em todos os distritos.

### Condições e situações de saúde autorreferidas

No módulo sobre condições e situações de saúde autorreferidas, o recadastramento levantou os seguintes dados: possuir plano privado de saúde, estar gestante, ser hipertenso ou diabético e estar acamado (tabela 4).

No que se refere a ter ou não plano de saúde privado, a tabela 4 mostra a baixa cobertura por algum tipo de plano de saúde da população residente em Foz do Iguaçu: apenas 20,4% proporção declararam ter algum plano de saúde privado. Essa proporção, no entanto, variou segundo distritos: em torno de 25% no Norte, Leste e Oeste e em torno de 14% no Sul e Nordeste.

Observa-se que 18,6% da população se autodeclararam hipertensos. Entre os distritos, essa proporção foi maior no Leste e Oeste (19,7% e 19%, respectivamente) e menor no distrito Nordeste (17,2%). Com relação ao diabetes, aproximadamente 7% da população informaram ser diabéticos. Mais uma vez, o distrito Nordeste apresentou o menor percentual (6,7%).

Durante o recadastramento, foram identificadas 523 gestantes, com idade variando de 14 a 45 anos. Ao relacionar o número de gestantes com o número de mulheres no período fértil (10 a 49 anos), observa-se que o número de gestantes por 1 000 mulheres foi mais elevado nos distritos Nordeste e Sul, os distritos de renda mais baixa. Esse mesmo indicador foi muito menor no distrito Oeste.

Para finalizar, entre a população residente recadastrada, foram identificados 265 indivíduos acamados, o que representa 4,0 pessoas acamadas por 1 000 habitantes no município. Essa proporção variou entre 3,4/1 000 no distrito Sul a 4,9/1 000 e 5,5/1 000 no Norte e Oeste, respectivamente (tabela 4).

## DISCUSSÃO

O recadastramento da população residente no município de Foz do Iguaçu reuniu informações de extrema relevância para subsidiar o planejamento da APS e também de outras áreas, como assistência social, trabalho e habitação. Em 3 meses de coleta de dados, foram visitados mais de 50 mil domicílios, com entrevistas realizadas em mais de 22 mil deles, totalizando pouco mais de 67 mil pessoas cadastradas. Foram levantados dados sobre condições da moradia, perfis socioeconômico e demográfico dos residentes e condições e situações de saúde autorreferidas.

**TABELA 4.** Indicadores da população recadastrada segundo distrito e condições e situações de saúde autorreferidas, Foz do Iguaçu (PR), Brasil, 2019

Variável	Distrito					Total
	Leste	Nordeste	Norte	Oeste	Sul	
Possui plano de saúde (%)						
Sim	24,5	14,3	26,5	24,6	13,8	20,4
Não	75,5	85,7	73,5	75,4	86,2	79,6
Hipertensão arterial (%)						
Sim	19,7	17,2	18,7	19,0	18,5	18,6
Não	80,3	82,8	81,2	81,0	81,4	81,4
Diabetes (%)						
Sim	7,6	6,7	7,3	7,3	6,9	7,2
Não	92,4	93,3	92,6	92,7	93,1	92,8
No. total	20 516	17 709	13 313	3 810	11 704	67 052
No. gestantes/1 000 mulheres	24,9	27,4	26,8	21,6	27,9	26,3
No. acamados/1 000 habitantes	3,7	3,6	4,9	5,5	3,4	4,0

***Fonte:*** Secretaria Municipal de Saúde de Foz do Iguaçu, Cadastre Já, 2019.

A opção pela estratégia de coleta de dados tipo varredura, ainda que não forneça uma amostra aleatória, mostrou-se muito apropriada para o trabalho de recadastramento realizado pelos ACS. O trabalho de campo foi facilitado e os ACS puderam conhecer a população adstrita ao seu território, como preconiza a Portaria 2 436 de 2017 do Ministério da Saúde, que estabeleceu a revisão das diretrizes para a Política da APS (9).

O esforço da Secretaria Municipal de Saúde na coleta de dados permitiu que os técnicos e os ACS se aproximassem da população e de sua realidade cotidiana. O recadastramento foi muito mais do que apenas um levantamento de dados: foi uma oportunidade para gestores e técnicos da administração municipal conhecerem o território e as principais características da população, contribuindo, assim, para aprimorar o planejamento das ações na área de saúde. Além disso, a experiência aprimorou a capacitação dos ACS — um elo importante entre a equipe de saúde e a comunidade — ao mostrar a importância da coleta de dados da população adstrita para a formulação de indicadores que subsidiem as ações e políticas de saúde em nível municipal (10).

Com relação às taxas de cobertura e de sucesso de realização da entrevista, observou-se que, nos distritos de mais baixa renda (Nordeste, Sul e Leste), a resposta ao recadastramento foi mais elevada. Nessas localidades, havia um número maior de equipes da ESF envolvidas e um número menor de domicílios fechados durante o recadastramento. Nos distritos Norte e Oeste, de maior renda, as taxas de sucesso das entrevistas foram muito baixas. Esse cenário é apoiado pela literatura, que evidencia maior cobertura da ESF em comunidades com menor nível socioeconômico e entraves de cadastro de segmentos mais abastados devido às dificuldades na realização de visitas domiciliares (11). No distrito Oeste, a proporção mais elevada de apartamentos (22,8%) também dificultou o acesso aos domicílios. Outras pesquisas domiciliares, como os censos demográficos, também relatam dificuldades de acesso aos domicílios de mais alta renda, sobretudo aqueles localizados em condomínios fechados (12).

Quanto aos resultados do recadastramento, ficaram evidentes as desigualdades sociais entre os distritos. Entretanto, quanto à distribuição etária, ficou claro que o processo de envelhecimento populacional ocorre de forma acelerada em todos os distritos; mesmo assim, ao analisar os valores populacionais absolutos, esses dados não apontaram nem um crescimento populacional expressivo no município no período mais recente, nem tampouco uma redução do número de habitantes como sugerem as estimativas municipais do IBGE (13). Nesse sentido, o recadastramento da população foi uma estratégia relevante para atualizar dados sobre a população residente e sua territorialização no município. Considerando que estimativas populacionais detalhadas por sexo e idade são essenciais para o planejamento e a execução das políticas, especialmente políticas de áreas sociais (14), os dados cadastrais da área da saúde deveriam ser mais frequentemente utilizados para a obtenção dessas estimativas.

Além de trazer informações para atualizar as estimativas do número de habitantes do município e levantar as características demográficas da população residente, o recadastramento produziu informações epidemiológicas fundamentais para o melhor planejamento das ações da APS no município. Um dado de grande relevância refere-se à baixa proporção daqueles que têm planos de saúde privados. Ficou claro que cerca de 80% da população do município dependem exclusivamente dos serviços públicos de saúde oferecidos pelo município, uma porcentagem maior do que a registrada para o estado do Paraná (15, 16). Considerando-se a dependência dos moradores de Foz do Iguaçu dos serviços públicos de saúde, o levantamento foi ao encontro das recentes mudanças na metodologia de financiamento da APS, antecipando-se ao esforço de cadastrar a sua população residente (2). Somando-se a isso, a experiência também contribuiu para a adoção de medidas de enfrentamento à pandemia de COVID-19 ao identificar grupos de risco para o agravamento da doença no município (portadores de doenças crônicas, pessoas acima de 60 anos, gestantes, puérperas e crianças abaixo de 5 anos) (17).

Uma limitação dos resultados do recadastramento está associada ao próprio processo de coleta de dados. Não se trata de uma amostra aleatória, mas de registros administrativos, que apresentam taxas de cobertura diferentes segundo distritos sanitários. A coleta foi feita por servidores públicos municipais (ACS e Agentes de Combate a Endemia, ACE) que tinham impedimentos legais para trabalhar fora do horário comercial (das 8h ao meio-dia e das 13h às 17h). Esse horário de trabalho favoreceu a coleta de dados em áreas de menor poder aquisitivo, onde havia maior presença de indivíduos nos domicílios no momento das visitas (distritos Nordeste e Sul). Por outro lado, prejudicou o avanço do recadastramento no centro comercial e administrativo do município, com maior nível socioeconômico e maior número de prédios (distrito Oeste). Desse modo, a inferência estatística para as características demográficas e epidemiológicas deve considerar os vieses no processo de coleta de dados. No entanto, dado o número elevado de domicílios visitados e com entrevistas realizadas, o município dispõe de uma base de dados atualizada sobre sua população residente, que deve ser utilizada no planejamento da APS.

Como lições aprendidas relatadas pelos gestores municipais, destacamos o envolvimento dos ACS no processo de visita e coleta de dados, que foi fundamental para promover a qualificação dos dados cadastrais da APS no município, e a utilização dos dados para reorganizar os serviços no atendimento da população residente e não residente no município durante o momento pandêmico. De fato, aprimorar os serviços de saúde para o atendimento mais adequado e eficiente da população residente, como também da não residente, é um desafio sempre presente para a gestão municipal em municípios fronteiriços como Foz do Iguaçu (18-20).

Em conclusão, a experiência de recadastramento da população em Foz do Iguaçu, como parte da rotina dos ACS, evidencia que a coleta periódica de dados pelos municípios para atender às normativas dos SIS pode e deve ser realizada. Como preconiza a Política de APS, recomenda-se que essa experiência seja reproduzida em outros municípios brasileiros, em especial nos fronteiriços.

## Declaração.

As opiniões expressas no manuscrito são de responsabilidade exclusiva dos autores e não refletem necessariamente a opinião ou política da RPSP/PAJPH ou da Organização Pan-Americana da Saúde (OPAS).
